# Suicide risk and mortality among patients with cancers of the digestive system: a systematic review and meta-analysis

**DOI:** 10.3389/fonc.2026.1655968

**Published:** 2026-01-26

**Authors:** Yebin Wang, Xiaotian Yang, Mengyao Song, Yipu Li, Dechao Jiao, Xueliang Zhou

**Affiliations:** 1 The First Clinical School of Medicine, Zhengzhou University, Zhengzhou, China; 2 Department of Interventional Radiology, The First Affiliated Hospital of Zhengzhou University, Zhengzhou, China

**Keywords:** digestive system cancer, meta-analysis, SMR, suicide, systematic review

## Abstract

**Background:**

Although significant strides have been made in cancer therapy, studies show that suicide rates among digestive cancer patients compared to the general population remain controversial, and specific risk factors are not clearly defined. Therefore, we conducted a systematic review and meta-analysis of the standardized mortality ratios (SMRs) in patients with digestive system cancer.

**Methods:**

PubMed, Embase, Web of Science and Cochran Library databases were searched for relevant articles up to June 2025. We assessed SMRs by cancer type, sex, cancer prognosis, time since diagnosis, geographic region and year of recruitment. We performed meta-analysis by random effects model and evaluated heterogeneity using I² statistic. Publication bias was performed by funnel plots, Edger’s and B egg’s tests.

**Results:**

12 cohort studies comprising 6763452 digestive cancer patients were eligible. The total SMR in this population was (SMR = 2.25, 95% CI = 1.89-2.66, P < 0.000). The SMRs were significantly increased for esophageal (SMR = 3.35, 95% CI = 2.37-4.75, P < 0.000), pancreatic (SMR = 3.87, 95% CI = 2.33-6.41, P < 0.000), stomach (SMR = 2.10, 95% CI = 1.81-2.43, P < 0.000), liver (SMR = 1.89, 95% CI = 1.38-2.59, P = 0.066) and colorectum (SMR = 1.57, 95% CI = 1.36-1.81, P < 0.000) cancers. Furthermore, the SMRs exhibit significant variations influenced by other contributing factors.

**Conclusions:**

Due to various suicide risk factors, the SMRs among digestive cancer patients surpasses that of the general population by more than double. Hence, heightened psychological support is warranted for these patients.

**Systematic Review Registration:**

https://www.crd.york.ac.uk/PROSPERO/view/CRD420261288056, identifier CRD420261288056.

## 1 Introduction

Due to the improvement of people’s living standards and the diversification of diet, more people are suffering from a variety of digestive system cancers (1). Gastric cancer (GC) ranks as the fifth most prevalent malignancy globally and holds the position of the fourth highest cause of cancer-related mortality worldwide (2). Despite the high incidence of GC, most patients are unfortunately diagnosed at advanced stages with poor prognoses due to the lack of distinguishing clinical indications (3). Once diagnosed with digestive system cancer, patients often endure substantial suffering. At the physical level, cancer treatments and interventions—including surgery, chemotherapy, radiotherapy, and hormonal therapy—exert profound impacts on the body (4). In addition, these treatments are frequently accompanied by psychological consequences. Patients diagnosed with digestive cancer commonly experience mental burdens such as anxiety, depression, physical distress, as well as social and economic stress (5). It has been reported that approximately 30% of patients with digestive system cancers develop adjustment disorders and depression, and that middle-aged individuals and those undergoing chemotherapy are at a higher risk of anxiety (6). In addition, suicide poses a significant public health dilemma (7). Compared with the general population, under the double burden of physical and mental stress, patients diagnosed with digestive cancers may be at heightened risk of suicidal behavior and the motivations behind such intentions can vary widely (8). Chen et al. reported that the SMR in esophageal cancer was 5.45 (95% CI = 4.66-6.35) in the U.S. general population (1975–2016) (9). Meanwhile, there are still studies that demonstrate no correlation between SMRs in patients with digestive tract cancer and the general population (10a). Therefore, regardless of the underlying reasons, suicidal behavior results in the loss of precious lives and inflict unimaginable suffering upon families and loved ones (11). Digestive system cancers seriously affect the diet and psychological condition of patients and in the past, the main approach was to use supportive psychotherapy to alleviate their symptoms for such patients, so it is necessary to conduct comprehensive management of psychological, spiritual and psychological treatment for cancer patients (12).

Moreover, there has been a conspicuous absence of meta-analyses investigating suicide rates in patients with digestive system cancer (13), alongside their associated risk factors, with many studies exhibiting shortcomings in terms of comprehensive literature reviews. Hence, the objective of this study was to quantify the SMRs among patients diagnosed with digestive system cancer in comparison to the general population. Furthermore, we undertook thorough subgroup analyses to assess the impact of specific risk factors on SMRs, including cancer prognosis, time since diagnosis, gender, and geographic region for providing targeted psychological support treatment for patients.

## 2 Materials and methods

### 2.1 Search strategy and study selection

We conducted a thorough literature review following the Preferred Reporting Items for Systematic Reviews and Meta-analyses guidelines (PRISMA guidelines) (14). A computerized search was carried out on PubMed, Embase, Web of Science, and Cochran Library databases to identify relevant studies published from the time of inception to March 2025. The search utilized the following terms to ensure comprehensiveness:(((((((stomach[Title/Abstract]) OR (gastric[Title/Abstract])) OR ((esophagus[Title/Abstract]) OR (esophageal[Title/Abstract]))) OR (((((colon[Title/Abstract]) OR (colonic[Title/Abstract])) OR (colorectal[Title/Abstract])) OR (rectum[Title/Abstract])) OR (rectal[Title/Abstract]))) OR ((((liver[Title/Abstract]) OR (hepatic[Title/Abstract])) OR (hepatocellular[Title/Abstract])) OR (intrahepatic bile duct[Title/Abstract]))) OR ((pancreatic[Title/Abstract]) OR (pancreas[Title/Abstract]))) AND ((((carcinoma[Title/Abstract]) OR (neoplasms[Title/Abstract])) OR (cancer[Title/Abstract])) OR (tumor[Title/Abstract]))) AND ((suicide[Title/Abstract]) OR (suicides[Title/Abstract])). A detailed outline of the process of study selection is presented in **Supplementary Table 1**.

### 2.2 Inclusion and exclusion criteria

The eligibility criteria for this review were determined based on the PICOS framework as follows:

(P) Population: Patients with digestive system cancers.
(I) Intervention: Diagnosed with a malignant tumor of the digestive system.
(C) Comparison/control: General population.
(O) Outcomes: Standardized mortality ratios.
(S) Study design: Observational study.

Only original studies conforming to the Population, Intervention, Control, and Outcome (PICO) framework were eligible for inclusion. Studies based on case reports and case series were excluded.

The inclusion criteria were as follows: (a) population characteristic: patients with digestive cancers: (b) outcomes: the statistical result is SMR of patients with cancer of the digestive system; (c) study type: all observational research (retrospective or prospective); (d) SMR shows no correlation between cancer patients and general population; (e) studies utilizing appropriate statistical methods for analyses and having sufficient data. The exclusion criteria were the following: (a) The number of patients is less than 100; (b) risk ratios other than SMR/HR; (c) The findings were specific to patients’ suicidal intentions. (d) confidence intervals ≠ 95%.

### 2.3 Study selection and data extraction

After removing duplicates, the initially identified articles underwent screening based on their titles and abstracts. If the abstracts seemed to meet the predetermined inclusion and exclusion criteria, a full-text assessment was conducted. Two reviewers (Wang and Yang) independently carried out the literature search and study selection using a standardized protocol. Any discrepancies between reviewers were resolved through consensus.

The following data were extracted from studies selected by two authors: (a) Study characteristics: author name, country, year of publication, study type, and year of inclusion. (b) Patient characteristics: total number of patients, gender, region. (c) Tumor characteristics: tumor type, tumor prognosis, years after diagnosis. Any discrepancies between authors were resolved through consensus.

### 2.4 Quality assessment

The Newcastle-Ottawa Standard (NOS) checklist was employed to evaluate the quality of the articles included in **Supplementary Table 2**. Each study underwent scoring by two independent reviewers, and any disparities were resolved through discussion and consensus with a third author.

### 2.5 Statistical analysis

As outcome measure, we used SMRs as RR estimates. In the absence of SMR data, we calculated the SMR values by comparing the observed number of suicides to the expected number in each country where the studies were conducted. Subsequently, we aggregated the observed and expected cases within each subgroup to determine the overall SMR. In order to minimize patient overlap, we opted for studies with the widest range of years of coverage and the largest sample size, and avoided overlapping follow-up years. Statistical analysis was conducted utilizing STATA v.12 software and R statistical software, the meta for packages, with a 2-sided 5% significance level. Meta-analytic pooling was executed exclusively when three or more studies were accessible. Meanwhile, we employed subgroup analysis and random-effects meta-regression to assess the potential changes in suicide rates among cancer patients according to cancer site, cancer prognosis, gender, geographic region, diagnostic period and the year of recruitment. Heterogeneity was assessed utilizing the Cochran Q-test and I^2^ statistics. A random-effects model was employed to amalgamate effect indicators in instances where I^2^ ≥ 50% and P < 0.05. Conversely, a fixed-effects model was utilized. Publication bias was examined when the analysis included ten or more studies.

## 3 Results

### 3.1 Study selection and characteristics

**Figure 1:**
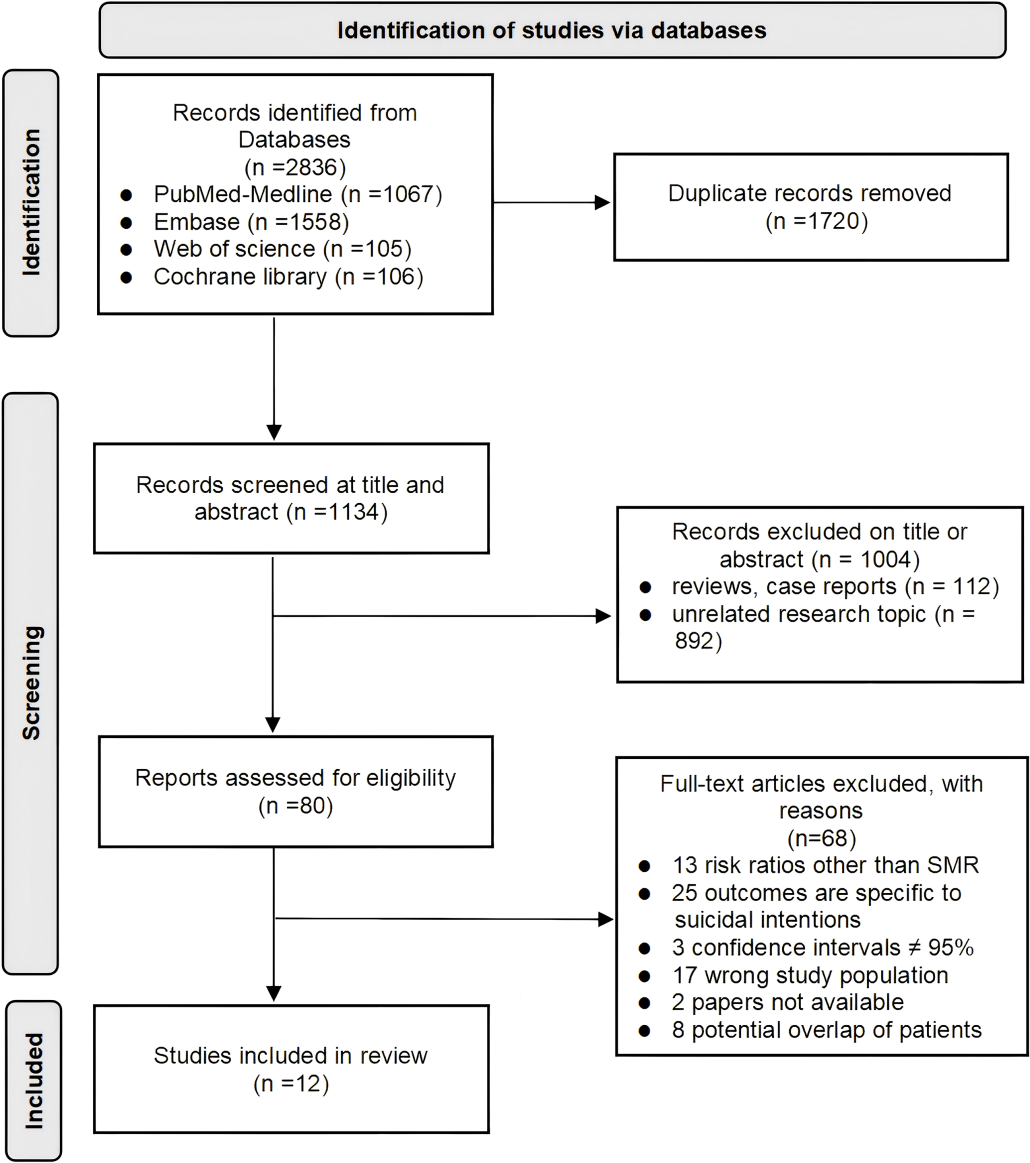
Flowchart of the literature selection.

We initially retrieved 2836 records, of which 1720 remained after excluding duplicates. Following title and abstract screening, 80 records were selected. Among these, 60 did not meet the inclusion criteria, resulting in 20 cohort studies for further analysis. Considering the potential overlap of patients in studies using the same database with recruitment periods and tumor types, we excluded eight records based on recruitment years, retaining a core set of 12 independent studies to mitigate the impact of patient overlap on meta-analysis outcomes. The flowchart of the literature selection process is depicted in **Figure 1**. In these twelve retrospective cohort studies, the results were consistently presented using standardized mortality ratios (SMRs), encompassing a total of 6,763,452 patients diagnosed with digestive system cancers, with follow-up periods ranging from 1960 to 2019. Among the total cohort of 6,763,452 samples of included studies, specifically, five studies incorporated both male and female participants, whereas only two studies were exclusively designed to examine female cohorts, for a total sample size of > 766,474 males and > 452,502 females. Geographically, the included studies spanned across Europe, the United States, and Asia, focusing on five major types of digestive system cancers: esophageal, hepatic, pancreatic, colorectal, and gastric cancers. Collectively, these studies sought to maximize representativeness by covering diverse geographic locations, balanced gender participation, and multiple types of digestive system cancers. The characteristics of the 12 retrospective cohort studies are presented in **Table 1**. All 12 studies were classified as high quality according to the Newcastle-Ottawa Standard (NOS).

**Table 1:** Characteristics of 12 retrospective cohort studies.

First author	Study design	Ethnicity	Cancer site	Country	Time of recruitment			Time diagnosis		Sex				Total population	No of suicide	SMR (95% CI)
										Male		Memale				
					Time	Number	SMR	With	Later	Number	SMR	Number	SMR			
15	Cohort study	White	(C)	USA	1988-2010	884529	1.33	NA		NA		NA		884529	1381	1.33(1.13-1.53)
16	Cohort study	White	(C)	Lithuania	1998-2012	19409	1.62	NA		9504	1.48	9905	2.15	19409	67	1.62(1.27-2.06)
17	Cohort study	Asia	(S)	Korea	2000-2016	434956	1.69	NA		NA		NA		434956	10143	1.69(1.59-1.79)
18	Cohort study	White	(C)(L)(S)	Polish	2009-2019	241040	2.007	NA		34001	2.77	19143	1.9	241040	NA	2.007(1.176-3.425)
19	Cohort study	Asia	(C)	Taiwan	2000-2010	96470	2.03	NA		NA		41697	1.09	96470	392	2.03(1.6-2.56)
10	Cohort study	White	(C)(L)(S) (E)(P)	USA	2000-2016	2715070	2.05	NA		NA		NA		2715070	3217	2.05(1.362-3.087)
20	Cohort study	White	(C)	USA	2000-2014	453774	2.08	572968	367551	NA		NA		453774	NA	2.08(1.74-2.47)
21	Cohort study	White	(C)(L)(S) (E)(P)	England	2015-2017	1002374	2.152	NA		NA		NA		1002374	508	2.152(1.369-3.382)
22	Cohort study	White	(S)	Norway	1960-1997	78413	2.21	NA		46467	2.15	31946	2.5	78413	94	2.21(1.5-3.14)
23	Cohort study	Asia	(C)(L)(S) (E)(P)	Korea	1993-2005	575249	2.292	151544	214325	385014	2.0	190235	2.35	575249	834	2.292(1.906-2.757)
24	Cohort study	White	(P)	USA	1973-2013	192395	5.39	NA		NA		NA		192395	NA	5.39(4.63-6.24)
9	Cohort study	White	(E)	USA	1975-2016	69773	69773	NA		NA		16108	2.47	69773	161	5.45(4.66-6.35)

C, colorectum; S, stomach; P, pancreas; L, liver; E, esophagus; F, women only; M, men only; NA, not available.

### 3.2 SMRs in patients with digestive system cancer

**Figure 2:**
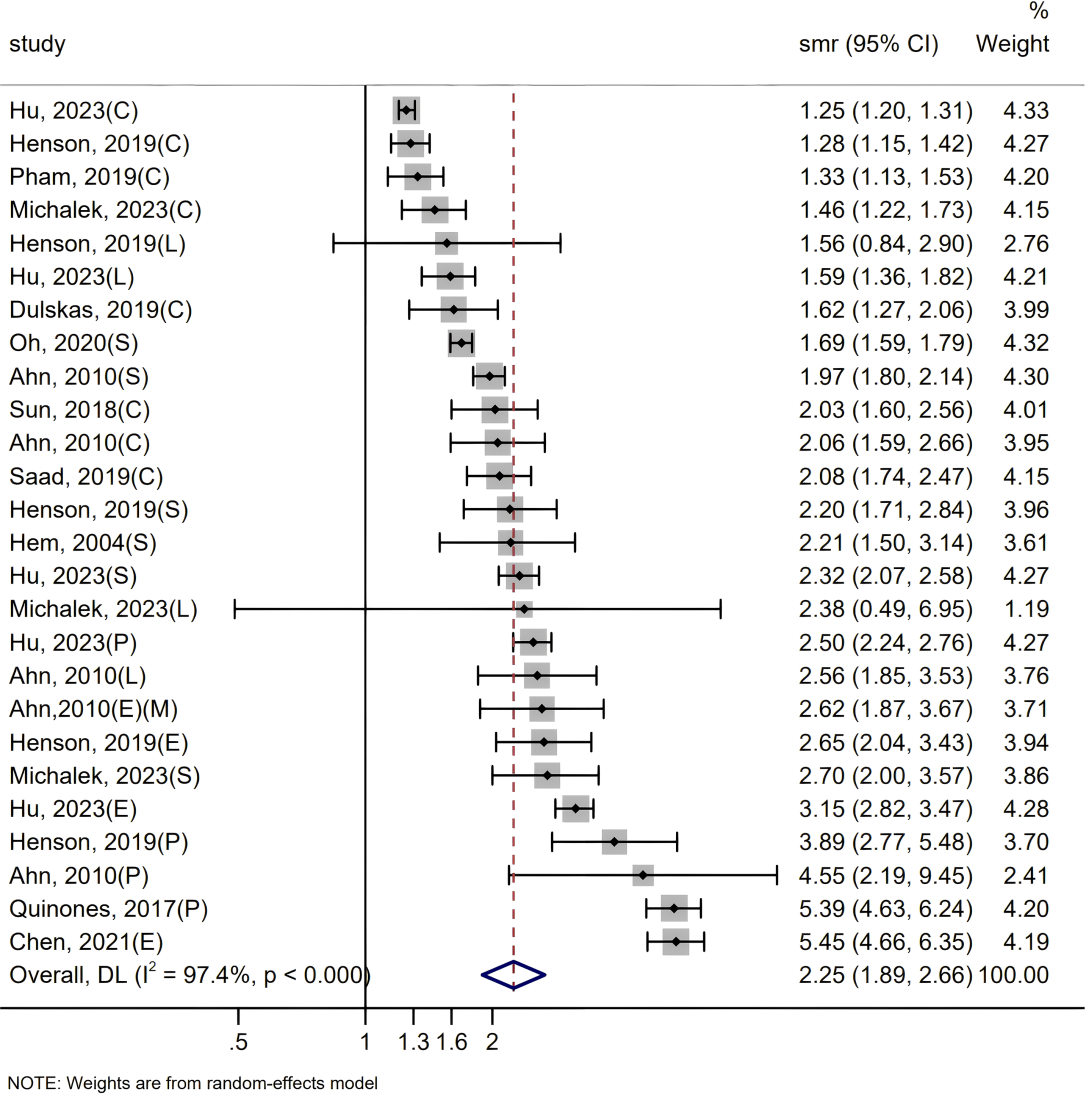
Since some articles contain SMRs of multiple digestive system cancers, individual cancer-specific SMRs are extracted and collectively analyzed in the forest plot. C, colorectum; E, esophagus; L, liver; S, stomach; P, pancreas; F, women only; M, men only.

**Figure 3:**
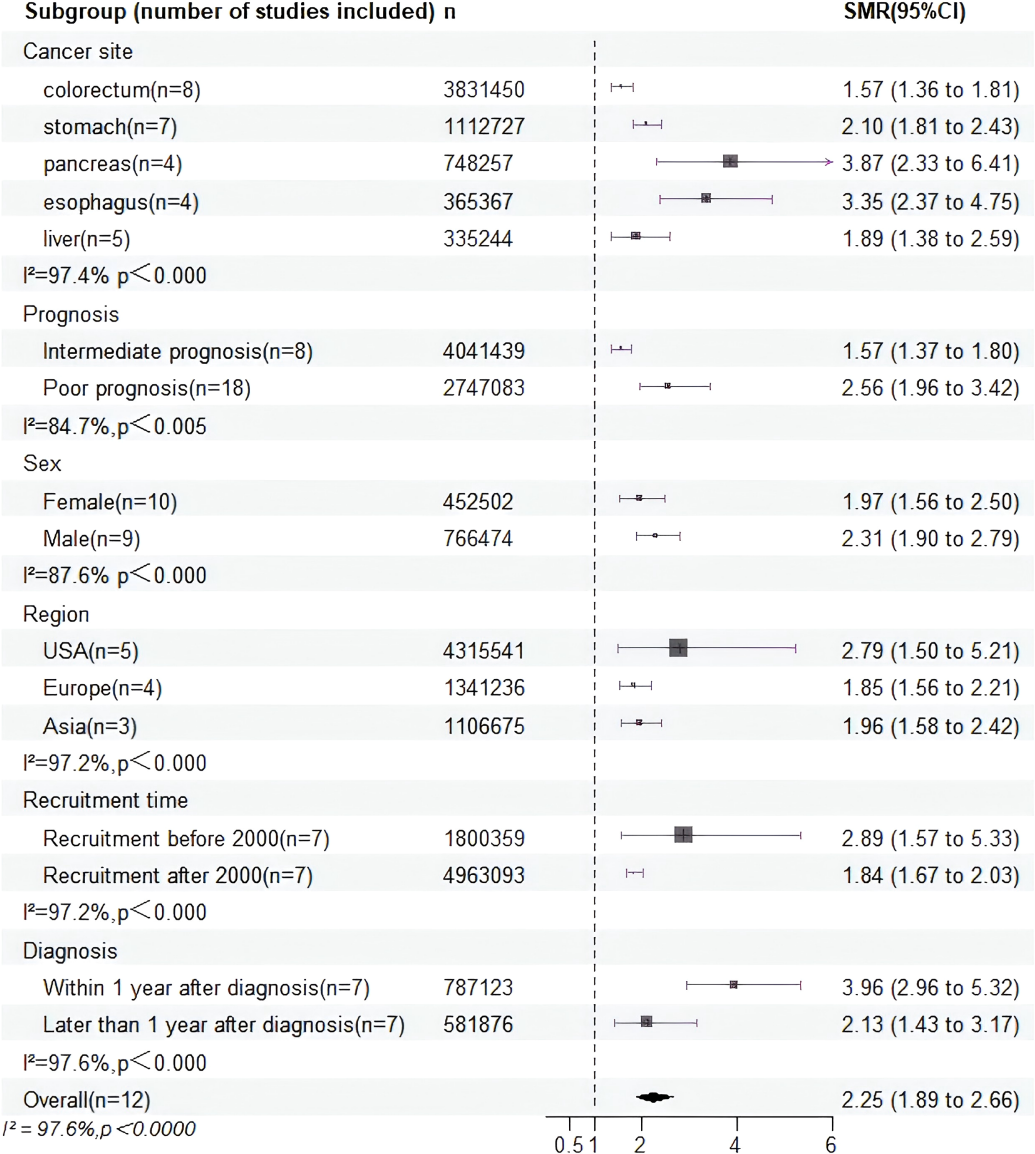
All subgroup analysis results were summarized.

Our comprehensive analysis revealed that the SMR among patients with digestive system cancer was 2.25 times higher than that of the general population (SMR = 2.25, 95% CI = 1.89–2.66, P < 0.000), accompanied by substantial heterogeneity (I² = 97.4%, P < 0.0001) in **Figure 2**. As the SMRs for patients with esophageal cancer, liver cancer, pancreatic cancer, gastric cancer, and colorectal cancer have been recorded together in certain studies. To obtain an integrated SMR for each cancer, we analyzed the results of these studies separately by cancer type in **Supplementary Figure 1**. The SMRs were significantly increased for esophageal (SMR = 3.35, 95% CI = 2.37-4.75, P < 0.000) (10b, 9, 21, 23), pancreatic (SMR = 3.87, 95% CI = 2.33-6.41, P < 0.000) (10, 24b, 21, 23), stomach (SMR = 2.102, 95% CI = 1.81-2.43, P < 0.000) (10, 17, 18b, 21–23), liver (SMR = 1.89, 95% CI = 1.38-2.59, P = 0.066) (10, 18b, 21, 23) and colorectum cancers (SMR = 1.57, 95% CI = 1.36-1.81, P < 0.000) (15, 20a, 10, 18b, 16, 19, 21, 23). To pinpoint potential sources of heterogeneity, we conducted further subgroup analyses. And the summary of subgroup analyses of suicide in patients is presented in **Figure 3** and **Table 2**.

**Table 2:** Summary table of meta-regression tests.

Co-variable	No of study	Sample	SMR	95%CI	*P* value	I²(%)
Cancer site			**2.25**	**1.89-2.66**	**<0.000**	**97.4**
Stomach	6	1112727	2.10	1.81-2.43	-	86.0
** Esophagus**	**4**	**365367**	**3.35**	**2.37-4.755**	**<0.000**	**93.0**
Liver	4	335244	1.89	1.38-2.59	0.066	58.4
** Colorectum**	**8**	**3831450**	**1.57**	**1.36-1.81**	**<0.000**	**88.4**
** Pancreas**	**4**	**748257**	**3.87**	**2.33-6.41**	**<0.000**	**95.7**
Sex			2.15	1.87-2.47	0.316	87.6
Women	7	452502	1.97	1.56-2.50	-	78.3
** Men**	**5**	**766474**	**2.31**	**1.90-2.79**	**<0.000**	**92.2**
**Prognosis**			**2.27**	**1.73-2.99**	**0.004**	**84.7**
Intermediate prognosis	8	4041439	1.57	1.36-1.81	-	0.0
** Poor prognosis**	**8**	**2747083**	**2.55**	**1.89-3.42**	**0.01**	**73.8**
**Time since diagnosis**			**2.98**	**2.14-4.16**	**0.014**	**97.6**
Later than 1 year	7	581876	2.13	1.43-3.17	-	91.2
** Within 1year**	**7**	**787123**	**3.96**	**2.96-5.32**	**<0.000**	**93.0**
Geographic region			2.30	1.69-3.12	0.453	97.2
Europe	9	1341236	1.85	1.56-2.42	-	0.0
** USA**	**10**	**4315541**	**2.79**	**1.50-5.21**	**<0.000**	**98.4**
** Asia**	**10**	**1106675**	**1.96**	**1.58-2.42**	**0.004**	**81.9**
Year of recruitment			2.30	1.69-3.12	0.153	97.2
After 2000	7	4963093	1.84	1.67-2.03	**-**	30.4
** Before 2000**	**5**	**1800359**	**2.89**	**1.57-5.33**	**<0.000**	**98.3**

Results are presented for the overall test as well as the comparison of each category compared with the reference category (depicted by a dash ‘–’). Variables with statistical significance are presented as emboldened. P value is the summary effect of each subgroup in the meta-analysis.

### 3.3 Cancer prognosis and time since diagnosis

The prognosis and time since diagnosis of digestive system tumors significantly influence patients’ emotional states and survival prospects. Given the generally poor treatment outcomes for most digestive system cancers, we categorized these cancers based on 5-year relative survival rates, defining them as having intermediate prognosis (5-year survival rates between 50–90%) or poor prognosis (5-year survival rates <50%) (25). Subgroup analysis by prognosis revealed notable differences in SMRs among patients (I² = 84.7%, P value for heterogeneity = 0.004) in **Supplementary Figure 2**. Specifically, for cancers with intermediate prognosis, such as colorectal cancer, SMR was reduced (SMR = 1.57, 95% CI = 1.36-1.81, P < 0.000). Conversely, for cancers associated with poor prognosis, including esophageal, liver, pancreatic, and gastric cancers, SMR was higher (SMR = 2.55, 95% CI = 1.89-3.42, P = 0.01), with lower heterogeneity (I² = 73.8%; P value for heterogeneity = 0.01, heterogeneity P < 0.05). Furthermore, significant statistical differences were also identified in the subgroup analyses stratified by time since diagnosis, as illustrated in **Supplementary Figure 3**. The included studies were categorized into two cohorts: those within the first year following diagnosis and those beyond the one-year post-diagnosis period. The SMR within the first year after diagnosis was significantly higher than more than one year after diagnosis, with high heterogeneity between studies (I² = 93%; P value for heterogeneity < 0.000). A multivariable meta-regression demonstrated that data pertaining to poor-prognosis cancers were redundant to suicide mortality within the first-year post-diagnosis, with poor prognosis emerging as the predominant contributing factor.

### 3.4 Gender, geographic region and year of recruitment

The SMRs among male patients (SMR = 2.31, 95% CI = 1.90-2.79, P < 0.000) diagnosed with digestive system cancer exhibited a pronounced elevation compared to that among female patients (SMR = 1.97, 95% CI = 1.56-2.50, P < 0.000) relative to their respective general populations, but the results did not reach statistical significance (P value for difference = 0.316; I² = 87.6%; P value for heterogeneity < <0.000) in **Supplementary Figure 4**. Furthermore, we observed that suicide mortality among patients with digestive system cancers was higher in the United States (SMR = 2.79, 95% CI = 1.50-5.21, P < 0.000) surpassed those in Europe (SMR = 1.85, 95% CI = 1.56-2.21, P = 0.463) and Asia (SMR = 1.96, 95% CI = 1.58-2.42, P = 0.004) in **Supplementary Figure 5**. Similar variations in suicide mortality were also identified across cohorts with different years of recruitment, as illustrated in **Supplementary Figure 6**. Patients recruited before the year 2000 (SMR = 2.89, 95% CI = 1.57-5.33, P < 0.000) exhibited noticeably higher suicide mortality compared to those diagnosed after 2000 (SMR = 1.84, 95% CI = 1.67-2.03, P = 0.196). Likewise, no statistically meaningful variations in SMRs were identified across gender (P value for difference = 0.316) and year of recruitment (P value for difference = 0.153), consistent with the absence of regional disparities.

The risk factors were also further categorized based on severity of suicide mortality (low, moderate, and high), as presented in **Table 3**.

**Table 3:** Summary table of risk factors by grade of suicide mortality.

Risk factor	Low risk	Intermediate risk	High risk
Cancer site	-	Colorectum, Liver	Pancreas, Esophagus, Stomach
Sex	-	Women	Men
Prognosis	-	Intermediate	Poor
Time since diagnosis	Later than 1 year	-	Within 1 year
Geographic region	-	Europe, Australia, Asia	USA
Year of recruitment	-	After 2000	Before 2000

Risk factors were stratified according to their SMR. As low risk, an SMR ≤ 1.50 was defined, risk factors with SMR 1.51–1.99 were included as intermediate and high risk was defined as SMR ≥ 2.00. No data were available for risk categories marked by a dash ‘–’.

### 3.5 Sensitivity analyses using all 12 eligible studies

We performed a sensitivity analysis utilizing a random-effects model on the 12 eligible studies identified in the systematic review, encompassing a cumulative sample size of 6,763,452 cancer patients in **Supplementary Figure 7**. From methodological, statistical, and clinical perspectives, heterogeneity is primarily associated with patients’ sex, geographic region, and disease prognosis. Notably, prognosis exerts a significant effect on suicide risk: the poorer the prognosis, the greater the physical, psychological, and economic burdens borne by patients, thereby increasing their susceptibility to suicide. The analysis produced coherent and stable findings. While sample overlap is conceivable, notably within SEER studies, the findings remain consistent with our initial analysis. Specifically, the standardized mortality ratio (SMR) was 2.25 (95% CI = 1.89–2.66) in the analysis based on 12 studies, compared to 1.95 (95% CI = 1.74–2.18) based on 9 studies.

### 3.6 Assessment of publication bias

A funnel plot displayed slight asymmetry concerning the pooled SMR across all 12 eligible studies, irrespective of potential sample overlap in **Supplementary Figure 8**. Nevertheless, both Begg’s correlation test and Egger’s regression test showed inadequate evidence to support the presence of publication bias (Begg’s test: P-value = 0.945; Egger’s test: P-value = 0.407).

## 4 Discussion

### 4.1 Summary and highlight

Through meta-analysis of 12 high-quality cohort studies, we have discovered that the overall SMR among patients with digestive system cancer is significantly higher than that of the general population, reaching more than twice the level. Especially, our study revealed a 3.9-fold elevation in suicide mortality among patients within the first year after cancer diagnosis and for those with a poor prognosis, their SMR also increases by 2.5 times. Previously, meta-analyses specifically focusing on patients with digestive cancers were nearly scarce, or there has been a lack of comprehensiveness, differences in sample size, and statistical capabilities (26). There was only one study on suicide among patients with digestive system cancers, and that was based on data from the SEER study (27). This study yielded valuable insights into the risk of suicide within this population. Nonetheless, this study was limited to patients in the United States and the follow-up period was too short. To comprehensively grasp the multiple factors contributing to suicide, it is imperative to obtain data from more countries and over an extended duration of time. In contradistinction, our meta-analysis transcended the confines of those previous analyses, encompassing a broader array of databases for the literature search. We diligently computed missing SMRs wherever data were accessible, augmenting our analytical rigor. Furthermore, we undertook supplementary analyses, including meta-regression, interaction analyses, and sensitivity analyses, thereby enriching the depth and breadth of our investigation.

### 4.2 Interpretation of results

#### 4.2.1 Cancer site

Variations in suicide mortality rates among patients with diverse types of digestive system cancer are striking. Statistical evidence revealed that the suicide incidence among colon cancer patients remained relatively subdued, merely 1.5 times that of the general populace (15b). This suggests a degree of psychological resilience among colon cancer patients, possibly linked to superior treatment outcomes and protracted survival periods (28). Conversely, the suicide prevalence among those grappling with gastric and hepatic malignancies markedly surpassed that of the general population, exceeding twice the norm (18, 21). Such elevated rates can be ascribed to the profound physical agony and compromised quality of life endured throughout the arduous treatment trajectories of gastric and liver cancers (29). The suicide incidence among esophageal and pancreatic cancer cohorts registered even more starkly, soaring three to fourfold above the general populace (10a). These malignancies typically portend poor prognoses and survival prospects, coupled with excruciating physical torment and precipitous deteriorations in quality of life during therapeutic interventions (30). Moreover, owing to their frequent late-stage diagnosis and limited therapeutic efficacy, this compounded sentiments of desolation and hopelessness among patients, amplifying their vulnerability to suicidal ideation (31). Hence, discerning the divergences in suicide frequencies among patients grappling with distinct cancer types assumes paramount significance for healthcare practitioners and medical teams alike.

#### 4.2.2 Prognosis and time since diagnosis

The association between cancer prognosis and elevated suicide mortality rates may reflect a causal relationship with tumor staging. Typically, as cancer progresses to advanced stages and the prognosis worsens, it leads to a decline in survival (32). Concurrently, patients facing late-stage cancer often endure heightened physical pain, psychological stress, and financial burdens, while also experiencing intensified feelings of despair and helplessness (33). In such circumstances, patients often necessitate more aggressive treatments, including chemotherapy, radiotherapy, or surgery, which may exacerbate physical discomfort and malaise, thereby amplifying psychological distress and fostering unstable emotions, culminating in suicidal (34).

Furthermore, the time since diagnosis emerges as another pivotal determinant of suicide mortality rates. Studies elucidate that cancer survivors within the initial year after diagnosis exhibit markedly escalated SMRs in comparison to those surpassing the one-year mark post-diagnosis (20, 23). This revelation intimates a plausible correlation between heightened suicide incidence among early-stage survivors and the unfavorable prognostic outlook associated with their malignancy. Throughout the first year subsequent to cancer diagnosis, individuals typically grapple with profound psychological strain and oscillations in emotional stability prompted by the diagnostic revelation (35). Moreover, they confront the necessity of acclimating to shifts in treatment modalities and lifestyle adjustments (36). Such adversities are poised to compound their psychological burdens and precipitate a profound sense of despondency, thereby engendering an augmented susceptibility to suicidal intention.

#### 4.2.3 Region and gender

The geographical region stands out as a significant determinant of suicide rates among patients grappling with digestive system cancer. Studies underscore a stark contrast in SMRs between cancer patients in the United States versus those in Europe and Asia (37). This discrepancy likely stems from disparities within the healthcare systems (10a). Although health care expenditure per capita is higher in the USA than in any other country, more than 37 million Americans do not have health insurance, and 41 million more have inadequate access to care (38). Previous studies using data from the early 2000s demonstrated that patients who were uninsured were more likely to present with late-stage disease and had worse short-term survival after cancer diagnosis in the United States (39). Consequently, many cancer patients in the U.S. face exorbitant medical expenses and treatment costs, imposing significant financial strain on both patients and their families, thereby heightening the suicide risk (40). Conversely, several European and Asian nations embrace universal healthcare systems, providing broader medical insurance coverage, and some even offer free or low-cost medical services (41). This ensures patients access to more comprehensive and affordable medical care, alleviating financial burdens during treatment and consequently reducing suicide risks. Furthermore, the impact of cultural and social beliefs on suicide rates cannot be overstated. American society tends to prioritize individual rights and freedoms (42), potentially fostering a greater inclination towards suicide as an escape from illness or adversity. Conversely, cultures in select European and Asian regions place a stronger emphasis on social cohesion and familial support, harboring more negative perceptions towards suicide, thus mitigating the propensity for patients to engage in suicidal behavior (43).

We’ve noticed that male cancer patients tend to face a significantly higher risk of suicide compared to their female counterparts (44). Gender distinctions remain highly relevant when considering the mental well-being and suicide susceptibility of cancer patients. Men might be more inclined to turn to aggressive or self-destructive behaviors as coping mechanisms for inner turmoil and hopelessness, further amplifying their chances of suicidal thoughts or actions (45). Conversely, although not statistically confirmed, the observed trend of lower suicide risk among female cancer patients could possibly be linked to their greater adaptability and stronger support networks, which offer them enhanced psychological resilience compared to their male counterparts (46, 47).

This study may be one of the most comprehensive and largest meta-analyses to date on the suicide mortality rates among patients with digestive system cancer. We provide important insights for better understanding the factors associated with suicide risk among patients with digestive system cancer. The findings of this research can prompt people to pay attention to the mental health of patients with digestive system cancer. Therefore, apart from focusing solely on medical treatment, it is essential to consider comprehensive support and intervention measures for mental health. This may involve providing psychological counseling services, establishing social support networks, and promoting psychological care education for patients and their families (48). Through collaborative efforts of multidisciplinary medical teams, we can provide them with better support and care.

## 5 Conclusions

Our results suggest that patients with digestive system cancer exhibit a significantly higher SMR compared to the general population, with suicide risks being closely associated with factors such as cancer type, sex, cancer prognosis, time after diagnosis, geographic region and year of recruitment. Especially, the elevated risk of suicide among patients with poor-prognosis cancers, as well as in countries where health insurance is not universal and illness can financially devastate not only the patient but also the entire family. Thus, enhanced attention and the implementation of more effective therapeutic and preventive strategies are imperative for this high-risk population. And the key limitation is that comprehensive and systematic treatment strategies for psychological disorders in cancer patients have not been established, highlighting the need for continued investigation.

## Data Availability

The original contributions presented in the study are included in the article/**Supplementary Material**. Further inquiries can be directed to the corresponding authors.

## Supplementary material

Supplementary Figure 1 Forest plot for subgroup analysis of cancer types.

Supplementary Figure 2 Prognostic subgroup analysis forest plot.

Supplementary Figure 3 Subgroup analysis of forest plot about post-diagnostic years.

Supplementary Figure 4 Sex subgroup analysis forest plot.

Supplementary Figure 5 Regional subgroup analysis forest plot.

Supplementary Figure 6 Years of recruitment subgroup forest plot.

Supplementary Figure 7 Sensitivity analysis plot.

Supplementary Figure 8 Funnel plot.
