# Supplementary figures and images for: Suicide risk and mortality among patients with cancers of the digestive system: a systematic review and meta-analysis
Source: Front Oncol. 2026 Jan 26;16:1655968. doi: 10.3389/fonc.2026.1655968 (PMC12883414; doi:10.3389/fonc.2026.1655968)

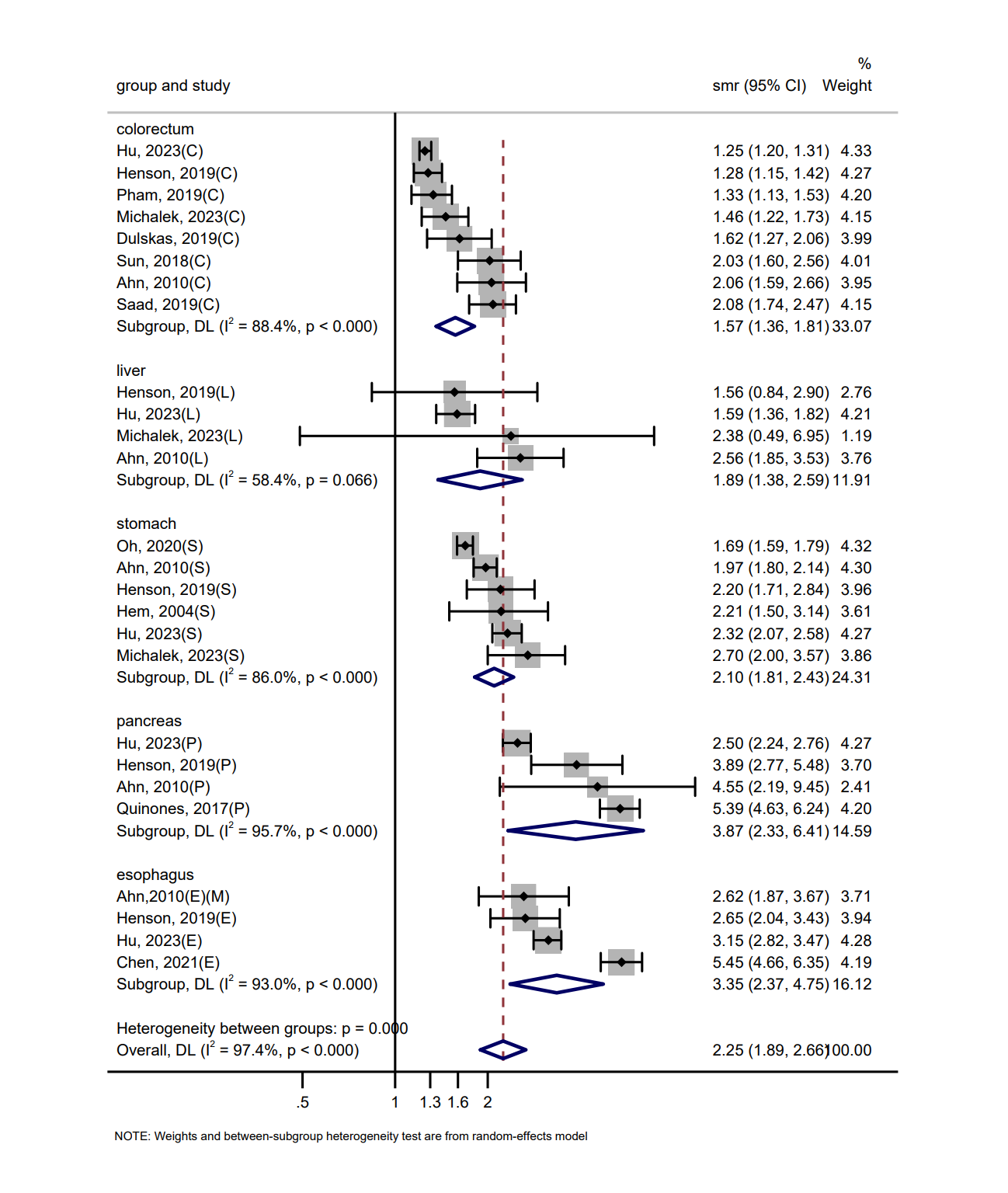

Supplement: Supplementary Figure 1 — Forest plot for subgroup analysis of cancer types. [file Image1.png]

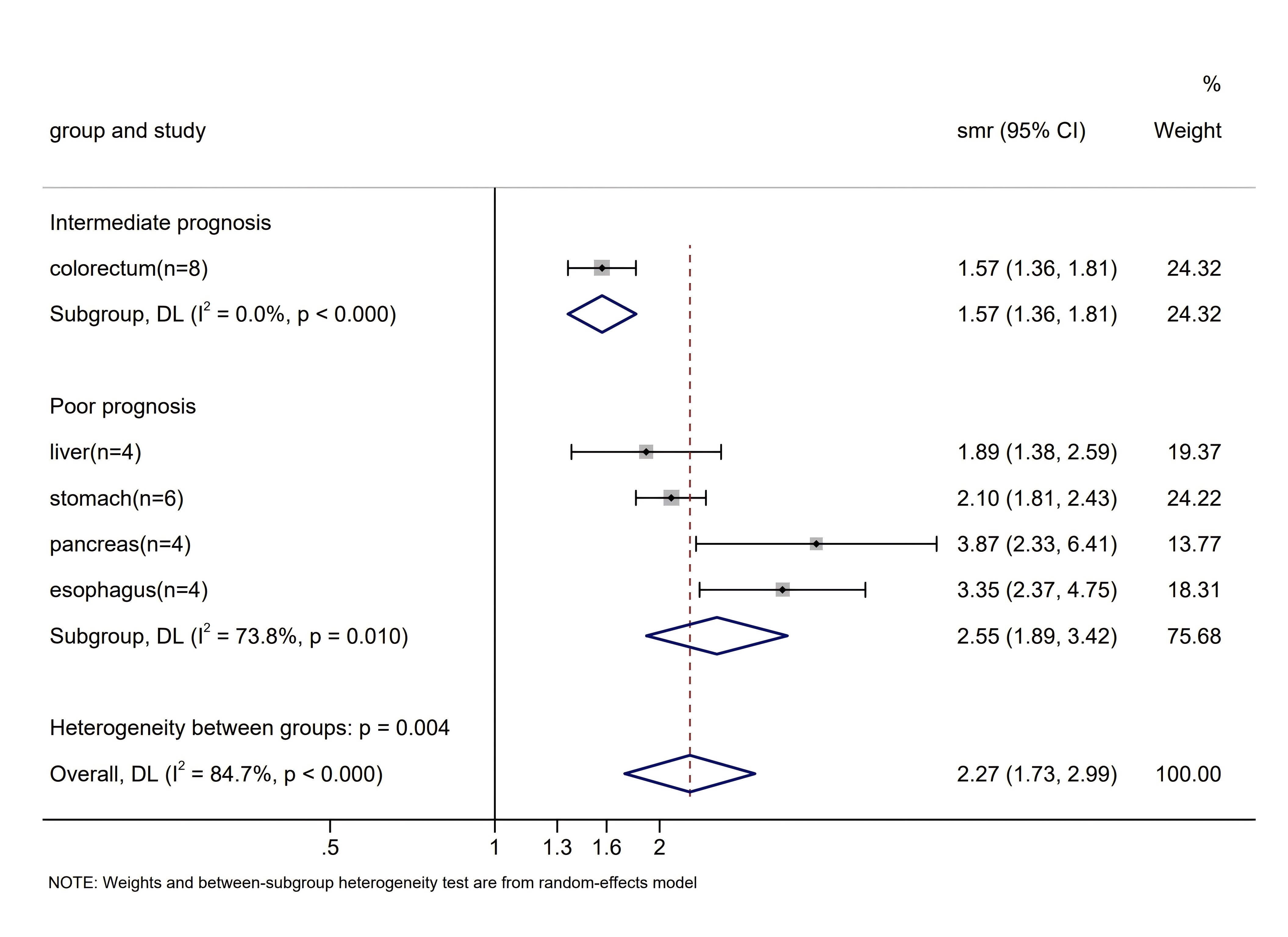

Supplement: Supplementary Figure 2 — Prognostic subgroup analysis forest plot. [file Image2.png]

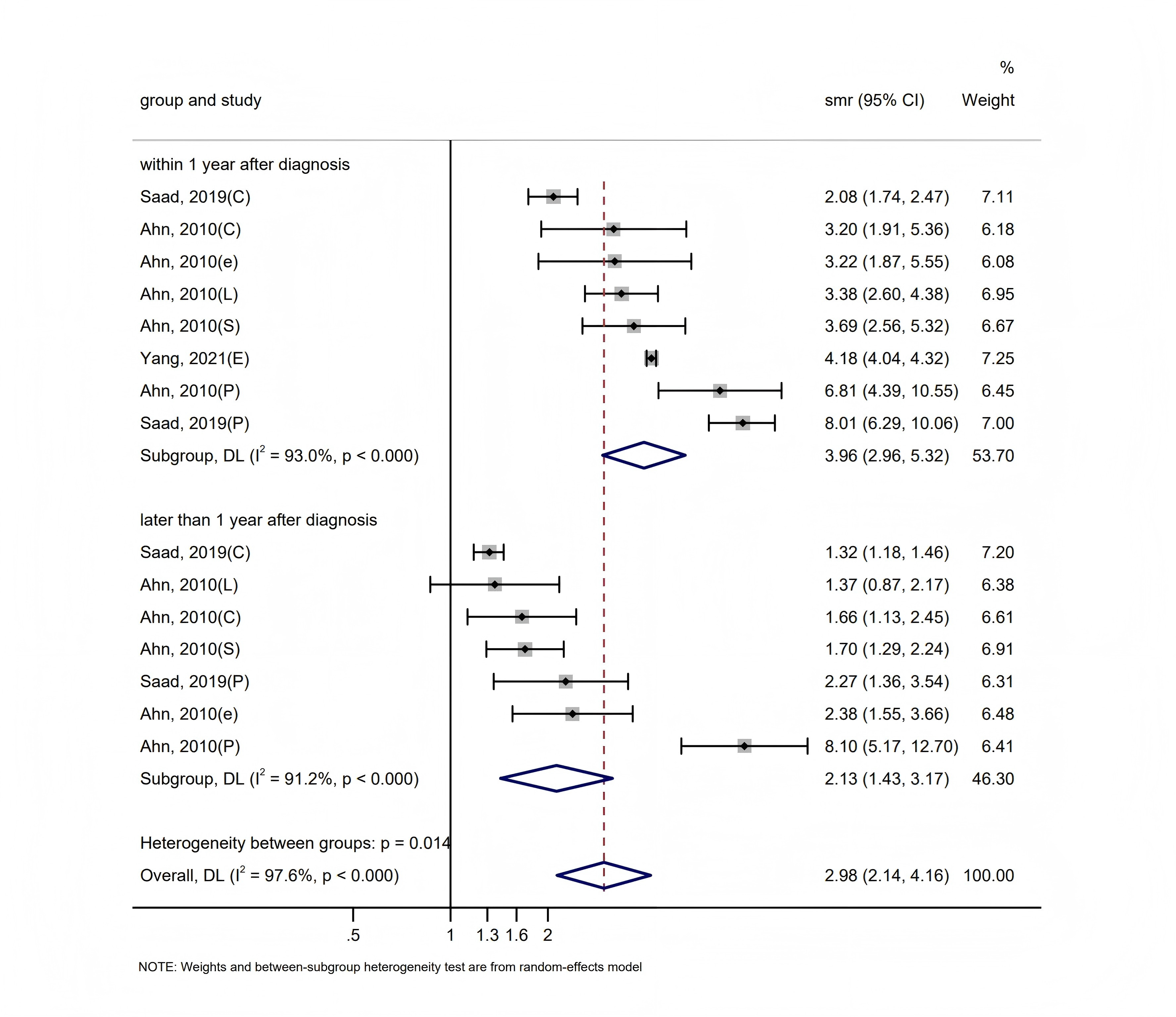

Supplement: Supplementary Figure 3 — Subgroup analysis of forest plot about post-diagnostic years. [file Image3.png]

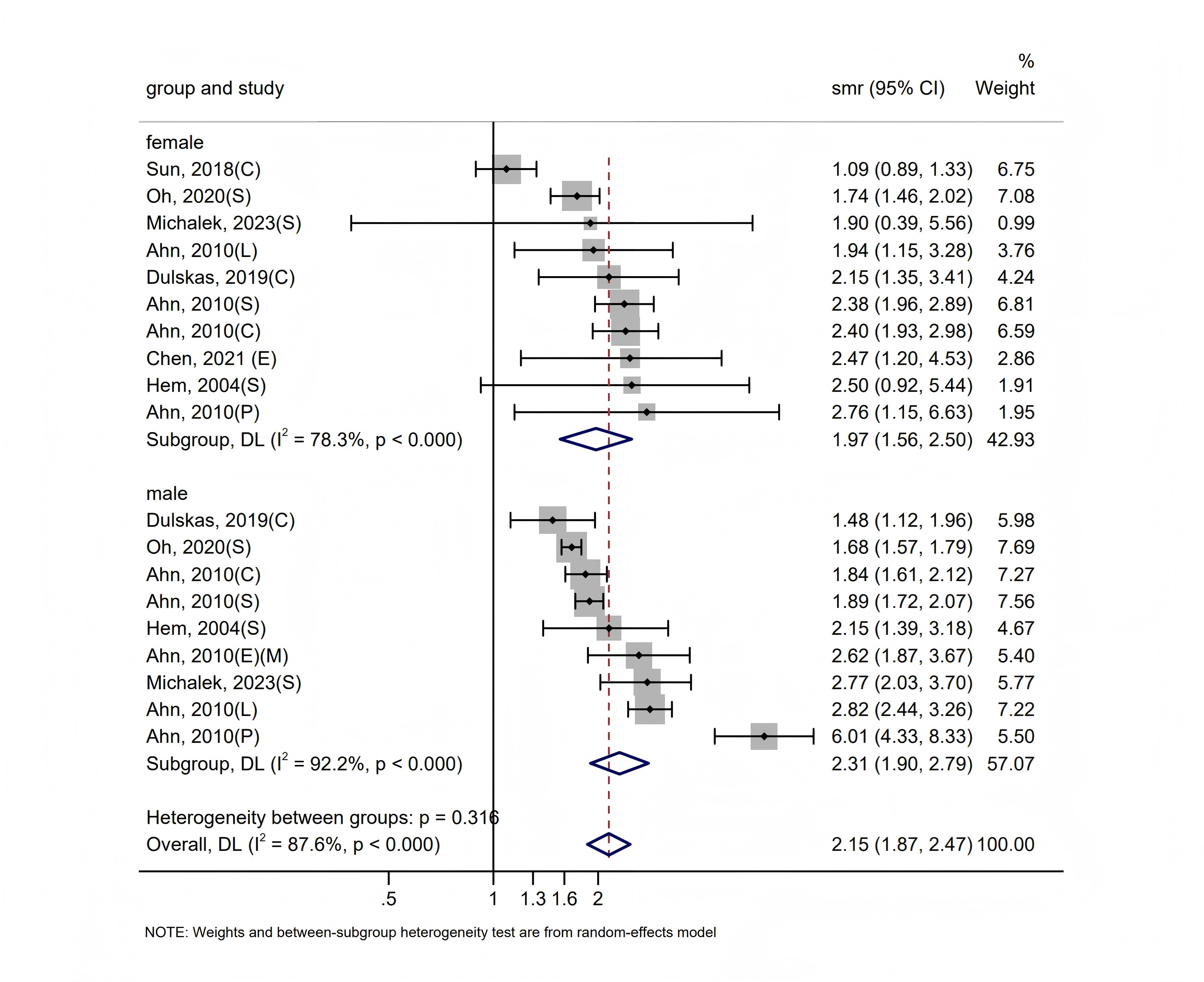

Supplement: Supplementary Figure 4 — Sex subgroup analysis forest plot. [file Image4.png]

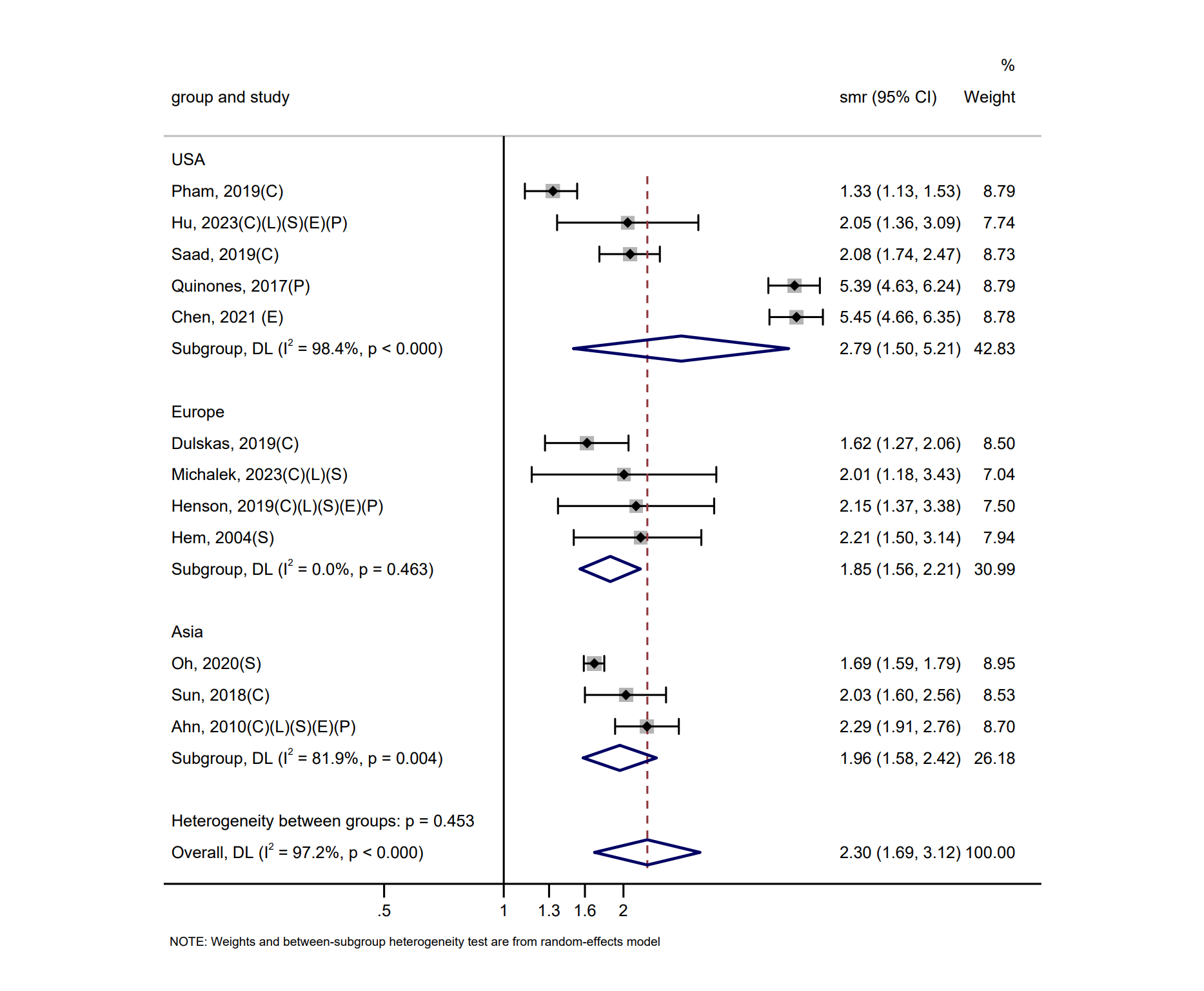

Supplement: Supplementary Figure 5 — Regional subgroup analysis forest plot. [file Image5.png]

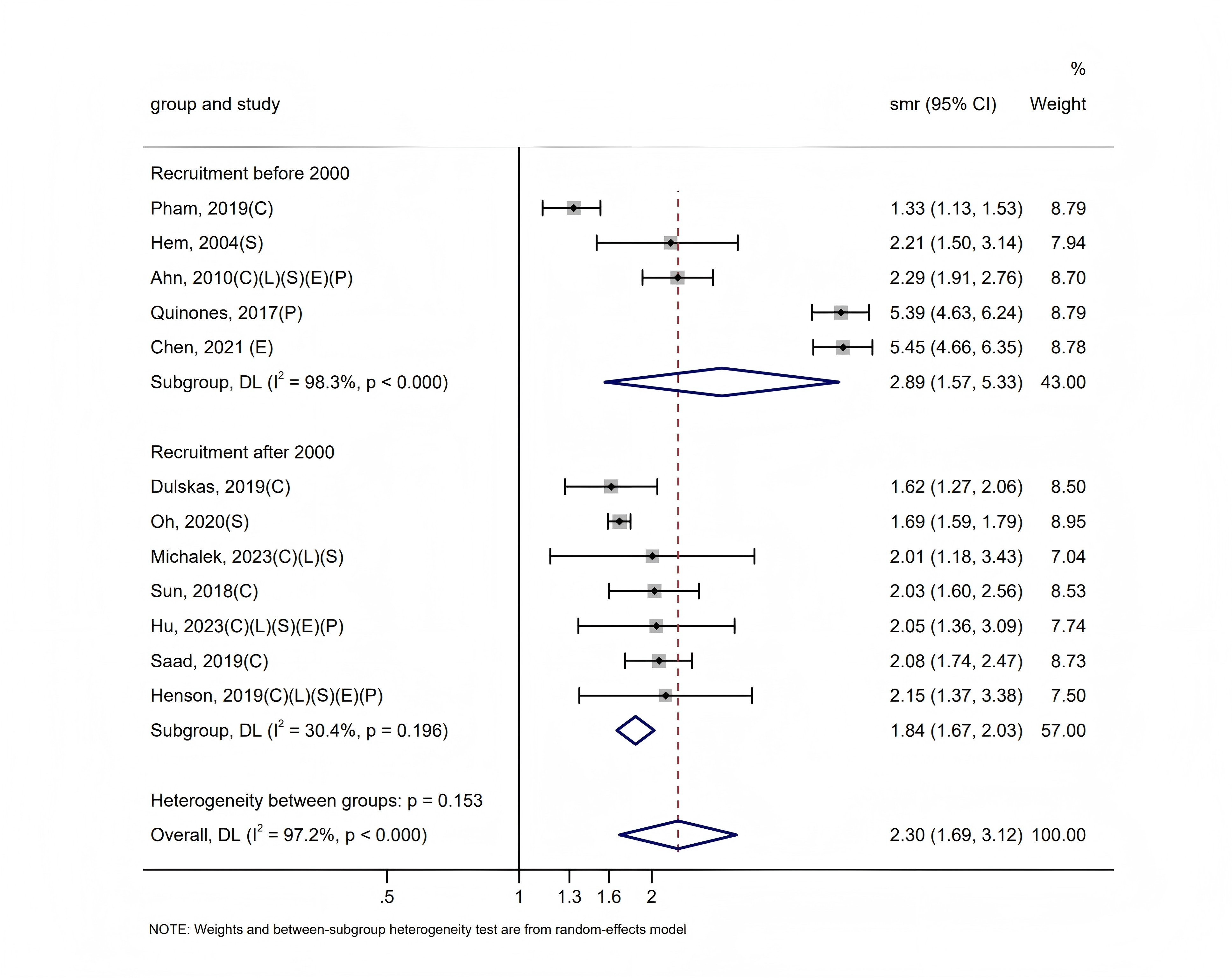

Supplement: Supplementary Figure 6 — Years of recruitment subgroup forest plot. [file Image6.png]

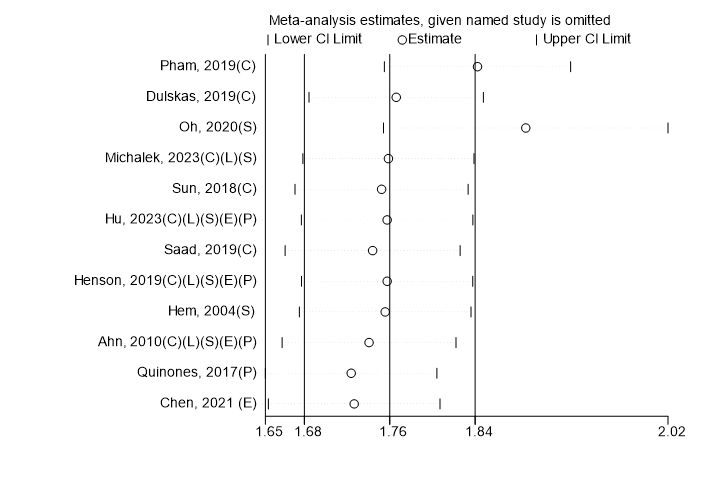

Supplement: Supplementary Figure 7 — Sensitivity analysis plot. [file Image7.png]

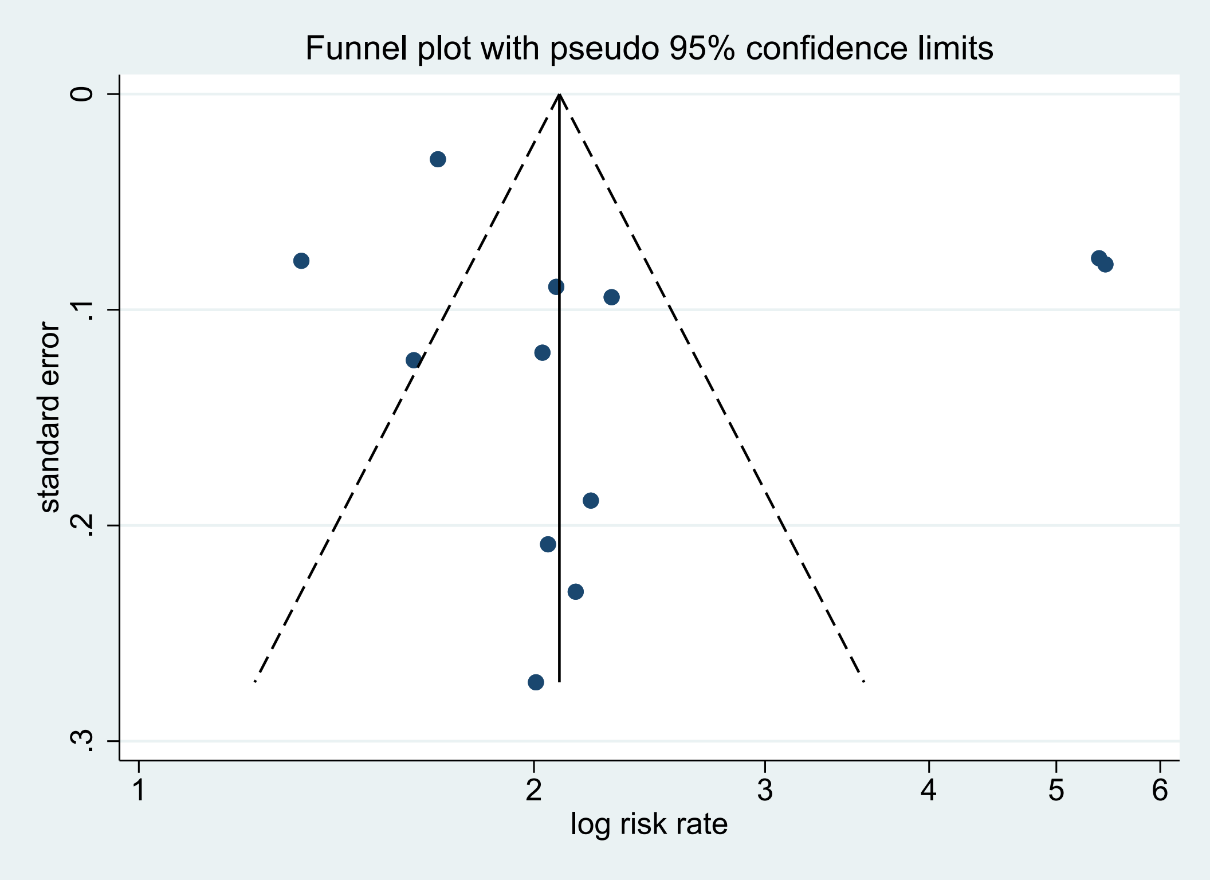

Supplement: Supplementary Figure 8 — Funnel plot. [file Image8.png]
