# Supplementary material for: Suicide risk and mortality among patients with cancers of the digestive system: a systematic review and meta-analysis
Source: Front Oncol. 2026 Jan 26;16:1655968. doi: 10.3389/fonc.2026.1655968 (PMC12883414; doi:10.3389/fonc.2026.1655968)
Supplement: Supplementary file 9 [file Table1.docx]

**Supplementary table 1:** The detailed search strategy (due to the complicated search procedure, only gastric cancer search terms are shown in detail, and other cancer search terms refer to gastric cancer).

| **Cancer type** | **Electronic databases** | **Search** | **Search strategy** | **Results** |
| --- | --- | --- | --- | --- |
| **stomach** | **PubMed** | #1 | (stomach [Title/Abstract]) OR (gastric [Title/Abstract]) | 387391 |
|  |  | #2 | (((carcinoma [Title/Abstract]) OR (cancer [Title/Abstract])) OR (neoplasm [Title/Abstract])) OR (tumor [Title/Abstract]) | 3533331 |
|  |  | #3 | #1 AND #2 | 147384 |
|  |  | #4 | (suicide [Title/Abstract]) OR (suicides [Title/Abstract]) | 83395 |
|  |  | #5 | #3 AND #4 | **154** |
|  | **Embase** | #1 | 'stomach':ab,ti OR 'gastric':ab,ti | 526421 |
|  |  | #2 | 'carcinoma':ab,ti OR 'cancer':ab,ti OR 'neoplasm':ab,ti OR 'tumor':ab,ti | 4827790 |
|  |  | #3 | #1 AND #2 | 205271 |
|  |  | #4 | 'suicide':ab,ti OR 'suicides':ab,ti | 102573 |
|  |  | #5 | #3 AND #4 | **200** |
|  | **Web of science** | #1 | TOPIC: (stomach) OR TOPIC: (gastric) | 15923 |
|  |  | #2 | TOPIC: (carcinoma) OR TOPIC: (cancer) OR TOPIC: (neoplasm) OR TOPIC: (tumor) | 246601 |
|  |  | #3 | #1 AND #2 | 7136 |
|  |  | #4 | TOPIC: (suicide) OR TOPIC: (suicides) | 8911 |
|  |  | #5 | #3 AND #4 | **6** |
|  | **Cochrane Library** | #1 | (stomach):ti,ab,kw OR (gastric):ti,ab,kw | 39213 |
|  |  | #2 | (carcinoma):ti,ab,kw OR (cancer):ti,ab,kw OR (neoplasm):ti,ab,kw OR (tumor):ti,ab,kw | 251650 |
|  |  | #3 | #1 AND #2 | 12120 |
|  |  | #4 | (suicide):ti,ab,kw OR (suicides):ti,ab,kwi | 8202 |
|  |  | #5 | #3 AND #4 | **9** |
|  | **total** |  | | **369** |
| **Esophagus** | **Pubmed** | #1 | (esophageal[Title/Abstract]) OR (esophagus[Title/Abstract]) | 49 |
|  | **Embase** | #1 | 'esophageal':ab,ti OR 'esophagus':ab,ti | 85 |
|  | **Web of science** | #1 | TOPIC: (esophageal) OR TOPIC: (esophagus) | 6 |
|  | **Cochrane Library** | #1 | (esophageal):ti,ab,kw OR (esophagus):ti,ab,kw | 7 |
|  | **total** |  |  | **147** |
| **Colon** | **Pubmed** | #1 | ((((colon[Title/Abstract]) OR (colonic[Title/Abstract])) OR (colorectal[Title/Abstract])) OR (rectum[Title/Abstract])) OR (rectal[Title/Abstract]) | 277 |
|  | **Embase** | #1 | 'colon':ab,ti OR 'colonic':ab,ti OR 'colorectal':ab,ti OR 'rectum':ab,ti OR 'rectal':ab,ti | 387 |
|  | **Web of science** | #1 | TOPIC: (colon) OR TOPIC: (colonic) OR TOPIC: (colorectal) OR TOPIC: (rectum) OR TOPIC: (rectal) | 29 |
|  | **Cochrane Library** | #1 | (colon):ti,ab,kw OR (colon):ti,ab,kw OR (colorectal):ti,ab,kw OR (rectum):ti,ab,kw OR (rectal):ti,ab,kw | 32 |
|  | **total** |  | | **725** |
| **Liver** | **Pubmed** | 1 | ((([Title/Abstract]) OR (hepatic[Title/Abstract])) OR (hepatocellular[Title/Abstract])) OR (intrahepatic bile duct[Title/Abstract]) | 425 |
|  | **Embase** | #1 | 'liver':ab,ti OR 'hepatic':ab,ti OR 'hepatocellular':ab,ti OR 'intrahepatic bile duct':ab,ti | 635 |
|  | **Web of science** | #1 | TOPIC: (liver) OR TOPIC: (hepatic) OR TOPIC: (hepatocellular) OR TOPIC: (intrahepatic bile duct) | 39 |
|  | **Cochrane Library** | #1 | (liver):ti,ab,kw OR (hepatic):ti,ab,kw OR (hepatocellular):ti,ab,kw OR (intrahepatic bile duct):ti,ab,kw | 49 |
|  | **total** |  | | **1148** |
| **Pancreas** | **Pubmed** | #1 | (pancreatic[Title/Abstract]) OR (pancreas[Title/Abstract]) | 162 |
|  | **Embase** | #1 | 'pancreatic':ab,ti OR 'pancreas':ab,ti | 251 |
|  | **Web of science** | #1 | TOPIC: (pancreatic) OR TOPIC: (pancreas) | 25 |
|  | **Cochrane Library** | #1 | (pancreatic):ti,ab,kw OR (pancreas):ti,ab,kw | 9 |
|  | **Total** |  | | **447** |
| **ALL Total** |  | | | **2836** |
