# Supplementary material for: Suicide risk and mortality among patients with cancers of the digestive system: a systematic review and meta-analysis
Source: Front Oncol. 2026 Jan 26;16:1655968. doi: 10.3389/fonc.2026.1655968 (PMC12883414; doi:10.3389/fonc.2026.1655968)
Supplement: Supplementary file 10 [file Table2.docx]

Supplementary Table 2: NOS Scores of all included studies. Four different authors evaluated study quality according to the Newcastle-Ottawa Scale (NOS). PMID: PubMed Identification number; DOI: digital object identifier. Newcastle-Ottawa Scale.

| **First Author** | **Year** | **PMID/DOI** | **Country** | **Time of recruitment** | **Wang** | **Yang** |
| --- | --- | --- | --- | --- | --- | --- |
| Pham | 2019 | 30027329 | USA | 1988-2010 | 8 | 9 |
| Dulskas | 2019 | 30617411 | Europe | 1998-2012 | 8 | 7 |
| Oh | 2020 | 31960609 | Asia | 2000-2016 | 7 | 8 |
| Michalek | 2023 | 10.1016/j.maturitas.2023.107785 | Europe | 2009-2019 | 9 | 7 |
| Sun | 2018 | 10.1002/pon.4891 | Asia | 2000-2010 | 8 | 8 |
| Hu | 2023 | 10.1001/jamanetworkopen.2022.51863 | USA | 2000-2016 | 9 | 8 |
| Saad | 2019 | 10.1002/cncr.31876 | USA | 2000-2014 | 8 | 9 |
| Henson | 2019 | 10.1001/jamapsychiatry.2018.3181 | Europe | 2015-2017 | 9 | 8 |
| Hem | 2004 | 15483032 | Europe | 1960-1997 | 8 | 7 |
| Ahn | 2010 | 20696665 | Asia | 1993-2005 | 7 | 8 |
| Quinones | 2017 | 10.1016/j.hpb.2017.02.049 | USA | 1973-2013 | 8 | 9 |
| Chen | 2021 | 34548616 | USA | 1975-2016 | 7 | 7 |
